# Focused Ultrasound Hyperthermia Augments Release of Glioma-derived Extracellular Vesicles with Differential Immunomodulatory Capacity

**DOI:** 10.7150/thno.46534

**Published:** 2020-06-12

**Authors:** Natasha D. Sheybani, Alec J. Batts, Alexander S. Mathew, E. Andrew Thim, Richard J. Price

**Affiliations:** 1 Department of Biomedical Engineering; University of Virginia, Charlottesville, VA 22908.; 2 Department of Radiology & Medical Imaging, University of Virginia, Charlottesville, VA 22908.

**Keywords:** Focused ultrasound, glioma, extracellular vesicles, exosome, cytokine

## Abstract

**Background**: Increasing evidence points to the critical role of extracellular vesicles (EVs) as molecular parcels that carry a diverse array of bioactive payloads for coordination of complex intracellular signaling. Focused ultrasound (FUS) hyperthermia is a technique for non-invasive, non-ionizing sublethal heating of cells in a near-instantaneous manner; while it has been shown to improve drug delivery and immunological recognition of tumors, its impact on EVs has not been explored to date. The goal of this study was to determine whether FUS impacts the release, proteomic profile, and immune-activating properties of tumor-derived EVs.

**Methods:** Monolayered murine glioma cells were seeded within acoustically transparent cell culture chambers, and FUS hyperthermia was applied to achieve complete coverage of the chamber. Glioma-derived EVs (GEVs) were isolated for characterization by Nanoparticle Tracking Analysis, cryo-electron microscopy and mass spectrometry. An *in vitro* experimental setup was designed to further dissect the impact of GEVs on innate inflammation; immortalized murine dendritic cells (DCs) were pulsed with GEVs (either naïve or FUS hyperthermia-exposed) and assayed for production of IL-12p70, an important regulator of DC maturation and T helper cell polarization toward the interferon-γ-producing type 1 phenotype.

**Results:** We confirmed that FUS hyperthermia significantly augments GEV release (by ~46%) as well as shifts the proteomic profile of these GEVs. Such shifts included enrichment of common EV-associated markers, downregulation of markers associated with cancer progression and resistance and modulation of inflammation-associated markers. When DCs were pulsed with GEVs, we noted that naïve GEVs suppressed IL-12p70 production by DCs in a GEV dose-dependent manner. In contrast, GEVs from cells exposed to FUS hyperthermia promoted a significant upregulation in IL-12p70 production by DCs, consistent with a pro-inflammatory stimulus.

**Conclusion:** FUS hyperthermia triggers release of proteomically distinct GEVs that are capable of facilitating an important component of innate immune activation, lending both to a potential mechanism by which FUS interfaces with the tumor-immune landscape and to a role for GEV-associated biomarkers in monitoring response to FUS.

## Introduction

Most, if not all, cell types are capable of producing extracellular vesicles (EVs) in order to mediate molecular transfer and complex cell-cell communication 1. EVs are phospholipid bilayer particles that can be distinguished on the basis of their biogenesis, functions and payloads into different classes, including apoptotic bodies, microvesicles and exosomes 2. Apoptotic bodies (50-5000 nm) are shed from dying cells during later stages of apoptosis. Microvesicles (200-1000 nm) arise from exophytic budding of the plasma membrane. Distinctly, exosomes (30-100 nm) arise endosomally through release of intracellular contents by multivesicular body (MVB) fusion to the plasma membrane 3,4. EVs carry rich payloads - such as proteins, RNAs, lipids and DNA - that are reflective of their parent cell, and this underscores their provocative potential role in intercellular signaling 1,5.

Tumor-derived EVs, notably exosomes, play a critical role in how tumors interface with the immune system, with the potential to potentiate both immunosuppressive and immunostimulatory mechanisms 6. Therapeutic interventions or modalities capable of inducing cellular stress such as chemotherapy 7-9, photodynamic therapy 7, microbubble-assisted ultrasound 10, irradiation 11, heat stress 12,13 and hypoxia 14 have been demonstrated to increase tumor-derived EV release. EV release under such conditions has also been implicated as a mechanism for tumor cell survival following radiation or chemotherapy 15.

Another disruptive technology with an emerging role in the landscape of anti-cancer therapy is focused ultrasound (FUS). FUS is a non-invasive, non-ionizing technique for focal acoustic energy deposition into tissues with submillimeter precision; acoustic parameters can be tuned to exert a variety of bioeffects in cells or tissues that range from thermal to mechanical in nature. In recent years, the continued promise of immunotherapy for cancer treatment has generated momentum for the advancement of FUS as a potentiator of immunotherapy in solid cancers 16,17. Moreover, a rich subset of FUS modalities assisted by the use of microbubbles, nanodroplets or sonosensitizers has been explored in the context of targeted therapeutic delivery or liquid biopsy applications 18-21; these studies have demonstrated a variety of use cases for FUS that include drug loading into EVs 20, delivery of EVs 18, controlled drug release from EVs 19, and enrichment of circulating cancer biomarkers via EV shedding 21.

To our knowledge, no studies have investigated the influence of FUS hyperthermia on EV release or profile to date. Thus, the goal of this study was to test the hypothesis that FUS hyperthermia augments the release of glioma-derived EVs (GEVs) and alters EV proteomic payload in a manner that is immunologically favorable. We herein characterize the size distribution, concentration, and proteomic profiles of EVs released from murine GL261-luc2 glioma cells following FUS hyperthermia. Finally, we examine IL-12p70 production by immortalized dendritic cells (DCs) following exposure to GEVs in order to determine whether FUS hyperthermia influences the capacity of GEVs for invoking a key signature of innate immune activation.

## Methods

### Cell culture

Luciferase-transduced GL261 murine glioma cells (GL261-luc2) obtained from the Woodworth Lab (University of Maryland) were cultured in complete growth medium containing high glucose Dulbecco's Modified Eagle's Medium (DMEM) supplemented with 10% Fetal Bovine Serum (FBS) + 1% non-essential amino acids (NEAA) + 1% sodium pyruvate (Gibco, Thermo Fisher Scientific) during standard cell passaging in T75 flasks. Cells were maintained at 37°C and 5% CO_2_.

### FUS hyperthermia treatments

**Figure 1:**
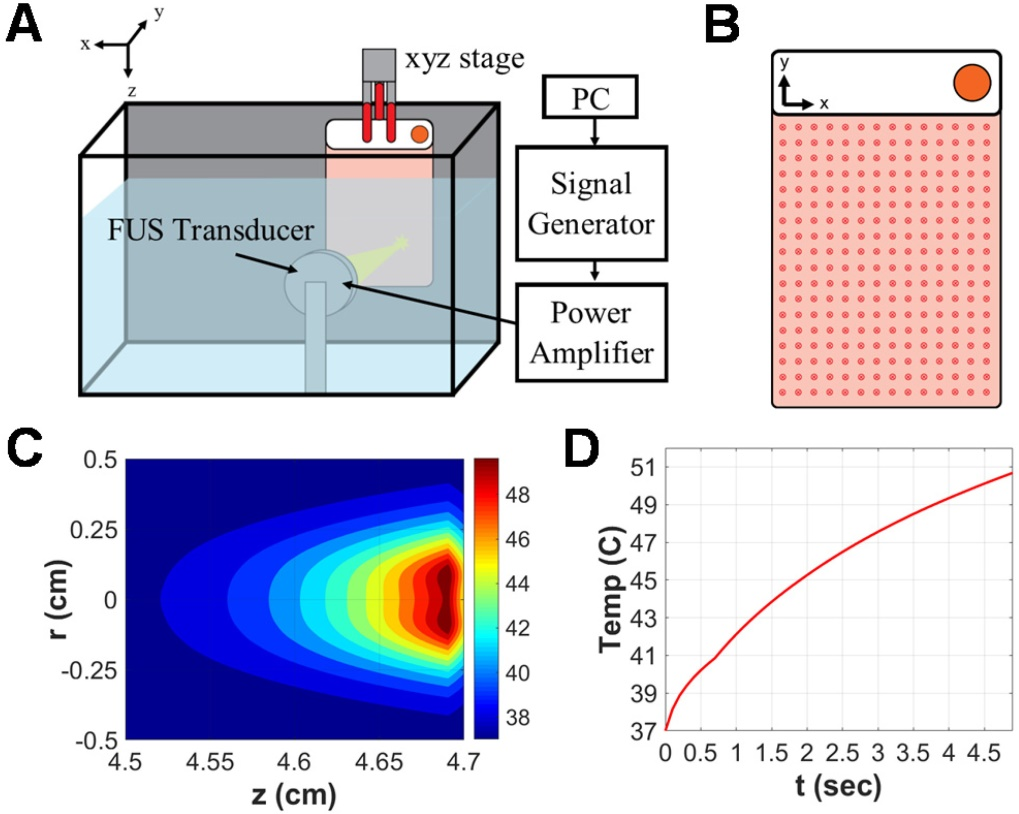
** Focused ultrasound hyperthermia application in PetakaG3 cell culture chambers. A.** Schematic representation of focused ultrasound system. All *in vitro* experiments were performed in a degassed water tank warmed to 37^⁰^C. For hyperthermia treatments, a 1.1 MHz single-element focused transducer was oriented directly across from the Petaka chamber. Position of the chamber with respect to the fixed transducer was controlled by a 3D linear motion controller. **B.** Representative layout of sonications applied to Petaka chamber. A 14x18 grid of sonications was applied to GL261-luc2 cells seeded within each chamber, with 5mm spacing between each insonation. **C.** Simulated spatial temperature profile for selected FUS hyperthermia parameters. The radius of hyperthermia (>40 °C) at the focus, as determined by simulation was approximately 2.5 mm. **D.** Simulated temporal evolution of focal temperature. Peak temperature at the focus was expected to reach 50°C at the surface (i.e. on the wall) of the Petaka for each sonication based on *in silico* predictions.

Approximately 24 hours prior to FUS exposure, GL261-luc2 murine glioma cells were seeded into acoustically transparent PetakaG3-HOT cell culture chambers (Celartia, Columbus, OH) in the presence of complete growth medium supplemented with 2% FBS. This closed-cell chamber setup was preferred over standard cell culture plates owing to its minimization of air pockets, contamination risk or acoustical interference and has been used in comparable studies 22,23. Chambers were handled according to manufacturer guidelines. Specifically, 80% confluent GL261-luc2 cells seeded in T75 flasks were transferred to Petaka chambers at a 1:2 sub-cultivation ratio (~4e6 cells per Petaka). On the day of FUS treatment, Petaka chambers seeded with the adherent monolayer of cells (~ 90% confluent) were loaded into a FUS system lined with a neoprene acoustic absorber (Figure 1A). Each chamber was acoustically coupled via degassed 37 °C water to a 64 mm spherical, single-element transducer (Sonic Concepts) operated at 1.1 MHz in continuous mode. An evenly spaced scan of 252 sonications (14x18 grid with 5mm spacing between insonations), each completed at 5W acoustic power over a 5s duration, was applied to achieve hyperthermia coverage over the entirety of each chamber (Figure 1B). Simultaneously, matched controls were incubated at 37 °C and 5% CO_2_ for the duration of FUS exposure (~42 minutes). Supernatant was harvested and clarified (300 × g for 10 minutes at 4 °C) from each chamber and its matched control 15 minutes following FUS exposure.

### FUS hyperthermia characterization

A derivative of the HIFU Simulator v1.2 software package (Joshua Soneson, US Food and Drug Administration) was utilized in MATLAB (The MathWorks, Inc., Natick, MA) to simulate the temperature rise associated with the selected parameters for *in vitro* treatment of a cell monolayer seeded in Petaka chambers 24. The model consists of propagation and heating modules, which solve the Khokhlov-Zabolotskaya-Kuznetsov (KZK) equation and Pennes' bioheat transfer (BHT) equation, respectively. For the Bioheat Transfer equation solution, specific parameters modeled for each material layer included: heat capacity (J/kg/K), thermal conductivity (W/m/K), perfusion rate (kg/m^3^/K), ambient temperature (37 °C), sonication duration (s) and duty cycle (%). For the KZK equation solution, specific parameters modeled for each material layer included: small signal sound speed (m/s), mass density (kg/m^3^), attenuation at 1 MHz (dB/m), power of attenuation vs. frequency curve, nonlinear parameter and material thickness (cm). Finally, transducer parameters included outer and inner radii (cm), focal depth (dm), frequency (MHz) and power (W). Manufacturer documentation was consulted for Petaka chamber specifications: dimensions -127.5±0.5 mm (x), 85.5±0.5 mm (y), 5±0.2 mm (z); volume of media - 20 mL; growth surface thickness - 0.9 mm; space between growth surfaces - 3 mm. Material composition (and associated acoustic attenuation coefficient) was modeled as crystal polystyrene.

Simulation of a single sonication using our established *in vitro* treatment parameters predicted an ~2.5mm focal radius of hyperthermia (>40 °C) as well as a peak focal temperature of 50 °C on the plastic wall of the Petaka chamber for each individual sonication (Figure 1C-D). Thermistor measurements capturing the temperature rise in media-filled void space between walls of the Petaka chamber revealed an average (± S.E.M.) peak temperature of 41.62 °C ± 0.63 °C. According to published manufacturer specifications, Petaka chambers have a maximum thickness of 5.2 mm, with the thickness of each wall being 0.9 mm; thus, the average temperature rise captured by thermistor measurements was consistent with that predicted to occur ~1 mm away from the peak temperature encountered at the Petaka wall.

These predictions of focal temperature were then confirmed by thermistor measurements. A Petaka chamber equally divided into nine uniform regions and positioned for sonication in a manner consistent with the previously described experimental setup. A thermistor (Yellow Springs Instruments, model #402) was fixed within the approximated center of each region of the 9-spot grid and each region was sonicated; peak temperature within each sonicated region was displayed via thermistor thermometer (Cole Parmer, model #8402-00) and averaged.

### Isolation of GEVs

GEVs were isolated by differential ultracentrifugation according to the detailed protocol set forth by Théry et al. 25. Briefly, clarified cell-culture supernatants were centrifuged at 2,000 × g for 20 minutes at 4 °C to remove dead cells. Supernatants were transferred to Beckman polycarbonate ultracentrifuge bottles and loaded into a 45 Ti rotor (Coulter-Beckman). Samples underwent ultracentrifugation at 9,000 rpm for 30 minutes at 4 °C to remove cellular debris and subsequently at 30,000 rpm for 80 minutes at 4 °C. Following this second ultracentrifugation step, the resulting EV pellet was resuspended in PBS, and 1 mL was harvested for Nanoparticle Tracking Analysis (NTA). A final washing and purification step was performed on the remaining resuspension via ultracentrifugation at 30,000 rpm for 80 minutes at 4 °C. The GEV pellet was isolated, resuspended in 50 µL of 1× PBS and stored at -80 °C for future use.

### Nanoparticle tracking analysis

EVs resuspended in 1 mL PBS were characterized in terms of size distribution and concentration. Samples were individually injected into the sample chamber of a NanoSight NS300 module (Malvern Panalytical, Westborough, MA) using sterile syringes (BD). Once liquid reached the tip of the nozzle, NTA Version 3.0 software was launched for video capture and particle movement analysis. Five independent measurements were taken for each sample and averaged to render mean, median and mode particle size as well as average particle concentration for each sample. Mode particle size is reported for all samples herein.

### Cryo-electron microscopy analysis

Purified samples were vitrified by standard methods for cryo-electron microscopy (cryo-EM). In brief, an aliquot (~3 µL) was applied to a glow-discharged, perforated carbon-coated grid (2/1-3C C-flats), blotted with filter paper, and rapidly plunged into liquid ethane. Low-dose images were recorded on a FEI Tecnai F20 transmission electron microscope operating at 120 kV, at a magnification of 29,000X or 62,000X with a pixel size of 0.37 nm or 0.18 nm, respectively, at the specimen level, and at a nominal defocus ranging from -1 to -3 µm. The grids were stored in liquid nitrogen, and then maintained in the microscope at -180 °C using a Gatan 626 cryo-holder. All images were recorded with a Gatan 4K × 4K pixel CCD camera.

### Mass spectrometry analysis

Each sample was reduced with 10 mM DTT then alkylated with 50 mM iodoacetamide in 0.1 M ammonium bicarbonate (both at room temperature for 30 minutes). The sample was then digested overnight at 37 °C with 0.1 µg trypsin in 50 mM ammonium bicarbonate. The sample was acidified with acetic acid to stop digestion and purified using magnetic beads (equal mixture Thermo Scientific Sera-Mag Speed Beads A and B) and finally evaporated to 15 µL for MS analysis. The LC-MS system consisted of a Thermo Scientific Q Exactive HF-X (or HF) mass spectrometer with a Thermo Scientific Easy Spray ion source connected to a Thermo Scientific 75μm × 15 cm C18 Easy Spray column (through a pre-column to wash - Thermo Scientific Acclaim Pepmap 75μm × 2 cm). 1 μg equivalent of the extract was injected and the peptides eluted from the column by an acetonitrile/0.1% formic acid gradient at a flow rate of 0.3 μL/min over 1 or 2 hours (A = 0.1% formic acid in water, B = 80% acetonitrile/20% Buffer A, gradient 2%B to 95%B). The nanospray ion source was operated at 1.9-2.4 kV. The digest was analyzed to determine peptide molecular weights followed by product ion spectra (Top10 HCD method) to determine amino acid sequence in sequential scans. The following instrument settings were used - cap temp 250⁰C, MS (1 microscan, AGC 3E6, max IT 60, 120K res), MS/MS (loop 10, 1 microscan, AGC 1E5, max IT 60, 30K res, isolation 2.0, NCE 27, intensity 2E3), dynamic exclusion 20sec, lock mass 445.12006. Raw data files were processed using Thermo Scientific Proteome Discoverer 2.2 using the Sequest algorithm and the following settings - parent 10ppm, fragments 0.02Da, tryptic, one missed cleavage, CAM cys fixed, Oxid Met dynamic, database Uniprot human/bovine or mouse/bovine. The resulting database search results were loaded into Proteome Software Scaffold 4.9.0 for filtering using the following custom settings - xcorr (+1>1.8, +2>2.0, +3>2.2, +4>3.0), Peptide Profit >60%, Protein Profit >90%. The resulting peptide FDR was generally ~0.5% and protein FDR ~1.5% (against reverse decoy database). The proteins were semi-quantified using spectral counts and normalized using the Scaffold Quantitative Value feature. Those proteins with two or more unique peptides were considered identified while those with one peptide were considered probable and to be confirmed manually as needed. All data and settings were contained within the RAW and Scaffold files. Data were further visualized and analyzed using Scaffold 4 (Proteome Software, Inc).

### Dendritic cell stimulation assay

GEVs were thawed and resuspended in 1 mL sterile PBS before sterile filtering (0.45 µm) immediately before DC stimulation. DC2.4 immortalized murine dendritic cells (MilliporeSigma, Temecula, CA) were passaged in complete growth medium (RPMI-1640 + 10% FBS + 1% HEPES buffer + 0.0054X β-mercaptoethanol) before seeding into 12-well plates at a density of 0.2e+06 cells/well. Upon reaching ~80% confluence, DCs were stimulated with sterile GEVs derived from either naïve GL261-luc2 cells or those treated with FUS. Twenty-four hours later, supernatant was harvested and clarified at 14,000 rpm for 10 minutes and stored at -80 °C until future use.

### ELISA assay

Harvested DC supernatants (as prepared above) were thawed and assayed in duplicate for IL-12p70 cytokine production (R&D Systems, Minneapolis, MN) according to the manufacturer's protocol. Optical density was determined using a plate reader set at 450 nm wavelength detection. Optical density readings at 540 nm were subtracted per the manufacturer's recommendation.

### Statistical Analysis

All statistical analyses were performed in Graphpad Prism 8 (Graphpad Software, Inc). A detailed description of statistical methods for each experiment is provided in the corresponding figure legend. All data are reported as mean ± standard deviation unless otherwise noted.

## Results

### Isolation and characterization of GEVs reveals greater release following in vitro FUS hyperthermia exposure, without significant impact on GEV size distribution

**Figure 2:**
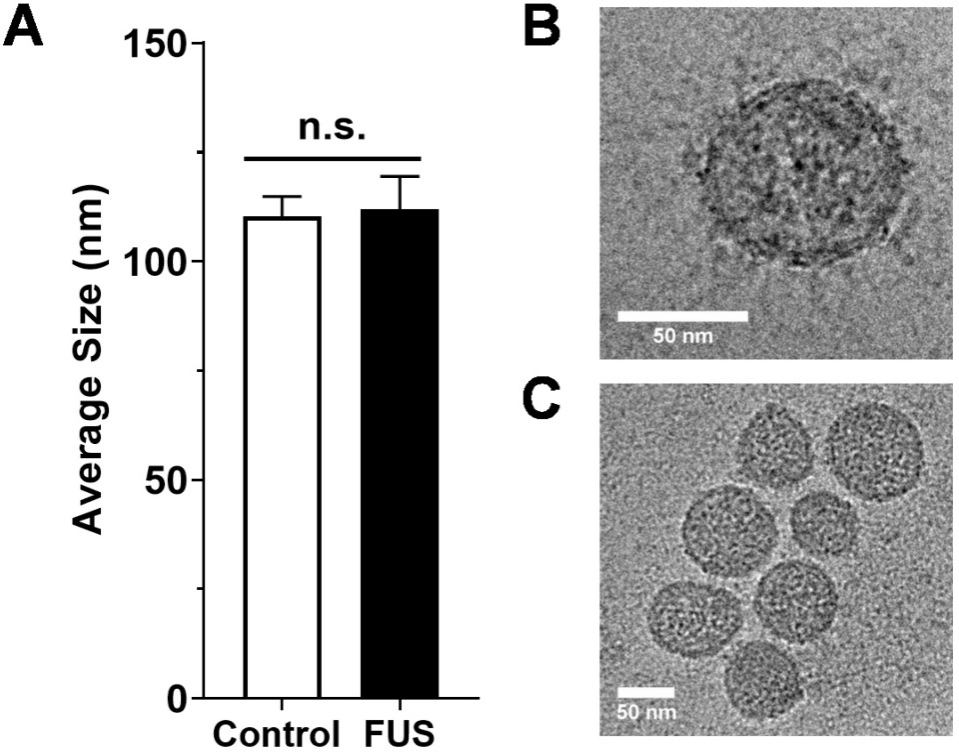
** Size characterization of GEVs. A.** Average size of GEVs isolated via differential ultracentrifugation according to mode diameter measured by NanoSight NTA. *n*=15-16 per group. **B.** Representative 62kx cryo-EM image of a 70.96 nm FUS hyperthermia-exposed GEV isolated by differential ultracentrifugation. **C.** Representative 29kx cryo-EM image of several GEVs treated with FUS hyperthermia. Particle diameters in this image range from 56.27 to 79.98 nm. Statistical significance assessed by unpaired two-tailed t-test. “n.s.” = not significant.

**Figure 3:**
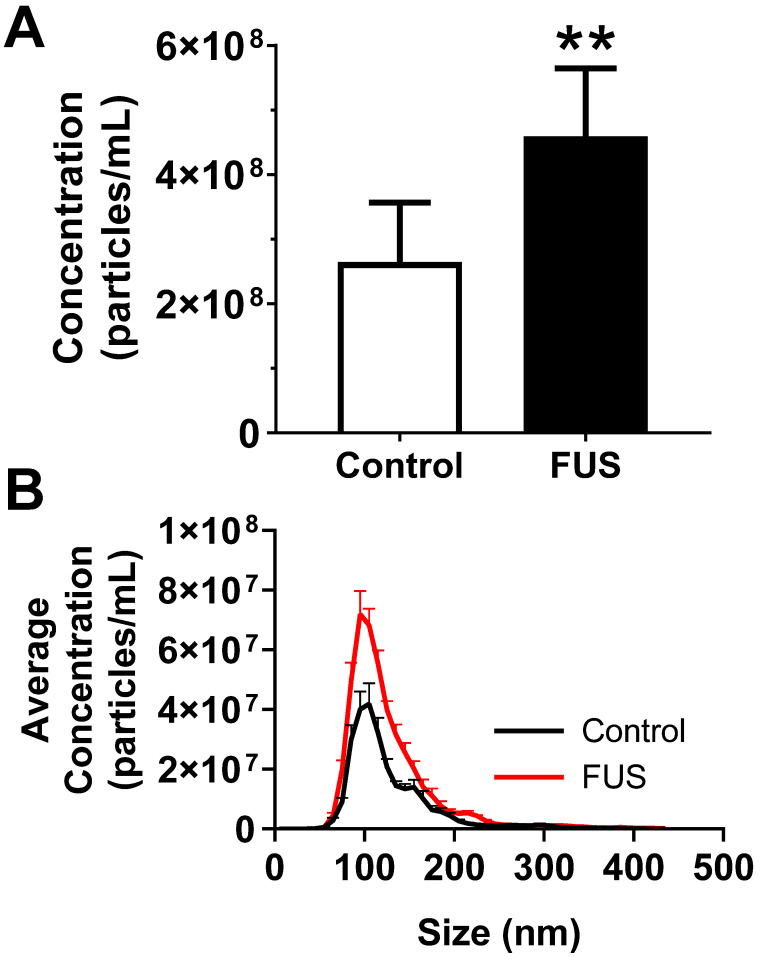
** FUS hyperthermia incites greater release of GEVs *in vitro*. A.** Overall concentration of EVs by Nanosight NTA profiling revealing a significant increase in EVs isolated 15 minutes following FUS hyperthermia exposure. **B.** Comparison of EV size distribution across experimental groups, indicating enrichment for particles ~110 nm in size on average. **p<0.01 vs. Control. *n=*8-9 per group. Statistical significance assessed by unpaired two-tailed t-test.

We first tested whether FUS hyperthermia stimulates the release or alters the size distribution of GEVs derived from GL261-luc2 cells. Fifteen minutes following FUS hyperthermia exposure, cell culture supernatants were clarified and prepared for EV isolation by differential ultracentrifugation. Following isolation and purification of GEVs, concentration and size distributions were determined by Nanoparticle Tracking Analysis (NTA). This analysis revealed that isolated GEVs were ~110 nm in mode diameter- consistent with an exosome-like identity; moreover, average size of GEVs was not significantly different between control and FUS hyperthermia conditions (Figure 2A). GEVs observed on cryo-EM had a shape, size and morphology consistent with those expected for a heterogenous population of EVs (Figure 2C-D). FUS hyperthermia exposure did not appear to qualitatively alter the morphology of released GEVs. By NTA analysis, it was further determined that exposure of GL261-luc2 cells to FUS hyperthermia elicited a statistically significant ~46% increase in GEV release compared to untreated controls (Figure 3A). Interestingly, comparison of GEV size distribution revealed no appreciable shifts with FUS; size distributions across both conditions were remarkably comparable despite the enrichment of average GEV concentration with FUS (Figure 3B).

### FUS hyperthermia exposure alters proteomic profile of GEVs

**Figure 4:**
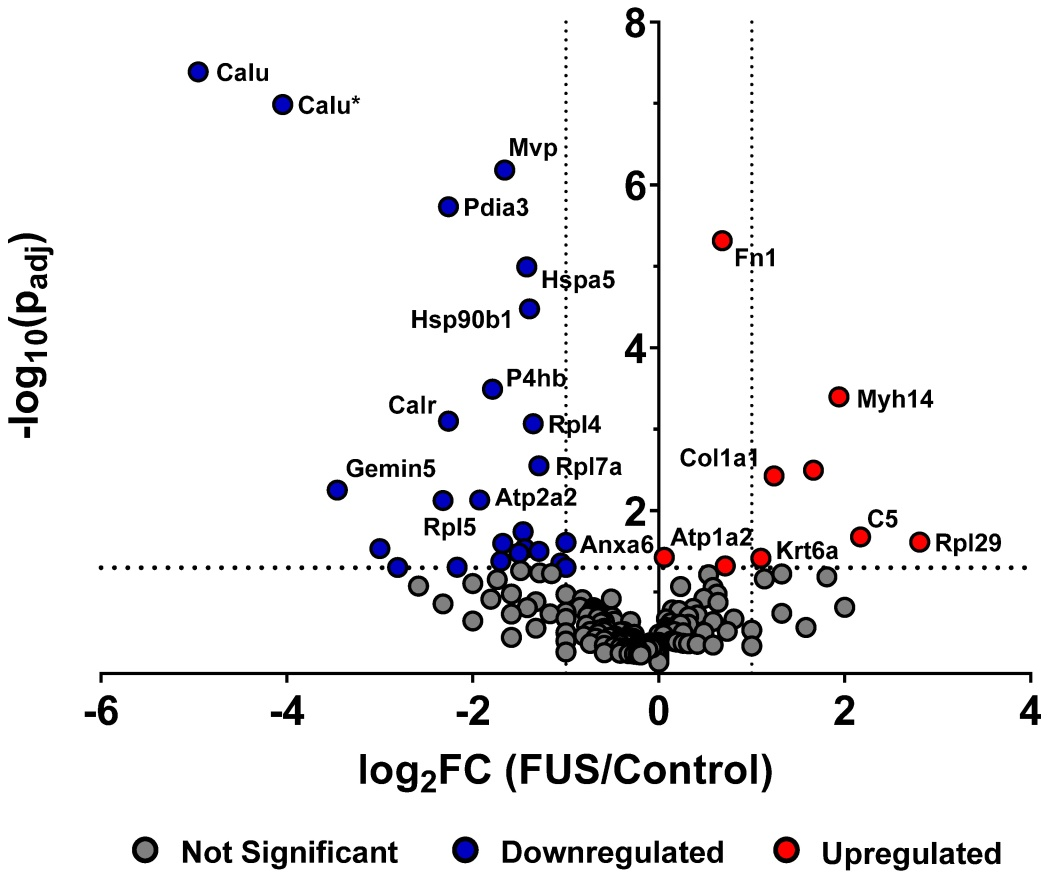
** FUS hyperthermia alters proteomic profile of GEVs.** Volcano plot depicting significantly regulated murine proteomic markers expressed by GEVs isolated via differential ultracentrifugation. Fold changes were tabulated as treated over control. Vertical black dotted lines denote fold change of ±2. Statistical significance was determined by Fisher's Exact Test with Benjamini-Hochberg correction. Horizontal black dotted line denotes p=0.05, which was considered the threshold for statistical significance. *n=*3 per group. Select proteomic markers are labeled with alternate protein IDs defined in the text and/or tables.

In order to determine whether FUS hyperthermia impacts the proteomic payload of released GEVs , purified control and matched treated GEVs isolated 15 minutes following conclusion of FUS treatment were analyzed by mass spectrometry. 1285 proteins were identified for control GEV samples, while 1336 were revealed for treated GEV samples. Comprehensive profiling for all bovine and murine peptides hits revealed that FUS hyperthermia significantly regulates a variety of proteins (Figure 4). Following curation of the raw data to account for “off-species” hits - i.e. manual exclusion of peptide hits that mapped explicitly to bovine origin - we noted that proteins including fibronectin (Fn1), myosin heavy chain 14 (Myh14), Keratin-6-alpha (Krt6a), collagen alpha-1(I) (Cola1a1), collagen alpha-1(II) (Col2a1), and complement C5 (C5) were significantly upregulated in GEVs originating from cells exposed to FUS hyperthermia versus their untreated counterparts. Conversely, proteins such as calumenin (Calu), endoplasmic reticulum chaperone (Hspa5), endoplasmin (Hsp90b1), calreticulin (Calr), major vault protein (Mvp), integrin alpha-6 (Itga6), annexin (Anxa6), among others were significantly downregulated on GEVs in the context of FUS hyperthermia. We provide a comprehensive list of significantly regulated murine GEV proteins in Table 1.

### GEV loading induces DC IL-12p70 production in a dose-dependent manner

**Figure 5:**
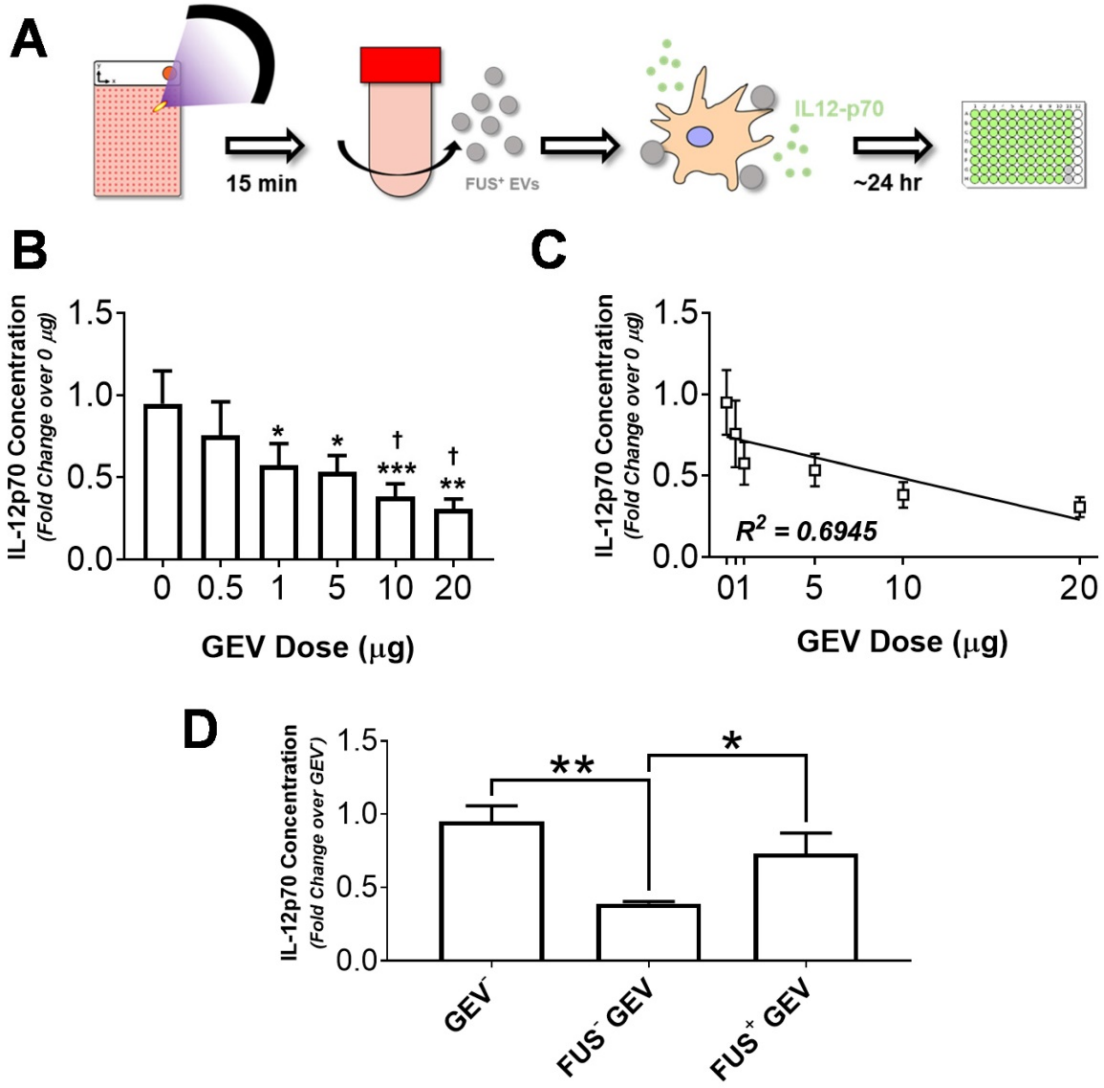
** Immortalized murine dendritic cells decrease IL-12p70 production in a GEV dose-dependent manner, and FUS hyperthermia-exposed GEVs promote a restoration of these levels. A.** Overview of experimental design. Briefly, GL261-luc2 cells were seeded in Petaka chambers. Supernatants from control or FUS hyperthermia-exposed cells were collected for GEV isolation by differential ultracentrifugation. Immortalized murine DC2.4 dendritic cells were exposed to GEVs for 24 hours, following which supernatants were collected for quantification of IL12-p70 production. **B.** Fold change in IL-12p70 production by DC2.4 cells following exposure to GEV doses ranging from 0 to 20 µg. *p<0.05, **p<0.01 vs. Control (0 µg GEV). ^†^p<0.05 vs. 0.5 µg GEV. 0-10 µg: *n*=4 per group, 20 µg: *n*=2. **C.** Linear regression analysis of GEV dose escalation data demonstrating a significantly nonzero slope (p<0.05, R^2^ = 0.6945) of -0.0254 ± 0.008423 and y-intercept of 0.7391 ± 0.07888. **D.** Fold change in IL-12p70 production by unstimulated DC2.4 cells, FUS^-^ GEV (1 µg dose), and FUS^+^ GEV (1 µg dose) groups. *p<0.05, **p<0.01 vs. group indicated. FUS^-^ GEV: *n*=2, GEV^-^, FUS^+^ GEV: *n*=4 per group. Statistical significance (for B,D) assessed by one-way ANOVA followed by Tukey multiple comparison correction.

In order to evaluate the capacity of GEVs from hyperthermia-stimulated cells to potentiate immune activation, we assessed cytokine production by immortalized murine DC2.4 dendritic cells following exposure to GEVs. DC2.4 cells were pulsed with various concentrations of GL261-luc2 GEVs for ~24 hours, following which supernatant was harvested and clarified for quantification of IL-12p70 production - a biomarker of DC maturation and Th1 differentiation - by ELISA (Figure 5A). Results indicated that for doses ranging from 0.5 to 20 µg, GEVs inhibit IL-12p70 production by DC2.4s in a dose dependent manner (Figure 5B). Significant reductions in IL-12p70 production were noted for all GEV doses greater than 0.5 µg relative to unstimulated DCs. Notably, approximately 2.5- and 3.1-fold reductions relative to basal DC2.4 IL-12p70 production were observed in the 10 µg and 20 µg dose groups, respectively. Linear regression analysis confirmed a strong correlation between IL-12p70 suppression and GEV dose, rendering a significantly nonzero slope (p<0.05, R^2^ = 0.6945) with fit line of slope -0.0254 ± 0.008423 and y-intercept of 0.7391 ± 0.07888 (Figure 5C).

### GEVs exposed to FUS hyperthermia mitigate IL-12p70 suppression

Given limited availability of GEVs from FUS hyperthermia treated cells (FUS^+^ GEVs), dosing studies were used to guide dose selection for subsequent studies. Accordingly, DC2.4 cells were pulsed either with 1 µg of FUS^+^ GEVs or an equivalent dose of GEVs from untreated cells (FUS^-^ GEVs) in subsequent stimulation experiments. Interestingly, we noted that for a matched dose of GEVs (1 µg), exposure of FUS^+^ GEVs significantly altered IL-12p70 production relative to naïve GEVs (data not shown). Specifically, a greater than two-fold decrease in IL-12p70 production was observed in the FUS^-^ GEVs group compared to unstimulated DCs, while a nearly two-fold increase in IL-12p70 production was brought about by FUS^+^ GEVs over their FUS^-^ counterparts (Figure 5D). While FUS^-^ GEV exposure rendered a significant decrease in IL-12p70 production relative to basal DC2.4 (GEV^-^) and FUS^+^ GEV conditions, these latter two conditions were not significantly different from each other (Figure 5D). IL-12p70 production by DC2.4 cells effectively returned closer to basal levels when cells were pulsed with FUS^+^ GEVs - suggesting the possibility for FUS hyperthermia exposure to potentiate recovery from the IL-12p70-suppressive activity intrinsic to GEVs.

## Discussion

In the present study, we report that FUS hyperthermia is capable of significantly altering the quantity and profile of EVs derived from murine GL261-luc2 glioma cells. To our knowledge, this is the first study to demonstrate the impact of FUS hyperthermia on EVs and the first to evaluate the capacity of FUS-exposed EVs to potentiate innate immunity. These findings suggest that hyperthermia, applied either alone or as an adjunct component to other focused ultrasound-based therapeutic approaches, could be useful for potentiating a robust innate immune response - specifically that of DCs - against gliomas. Given the emerging role of FUS as an adjunct to immunotherapy in brain malignancies 16,17, our study generates timely insights into the largely untapped mechanistic underpinnings of FUS that could exist in the context of EV biology.

We observed that near-instantaneous hyperthermia induction in monolayered GL261-luc2 cells gave rise to significantly elevated GEV concentration within 15 minutes following conclusion of FUS treatment. While we took a holistic approach to addressing our hypothesis by remaining agnostic to GEV subtype, the size distribution data we collated via NTA analysis suggest that the most highly enriched population within our GEV isolates falls within the expected size range of exosomes. Moreover, our size distribution findings did not support the notion that FUS hyperthermia may be stimulating the preferential release of vesicle subtypes distinct from those released in the control setting. Our observation of EV augmentation with FUS is consistent with those made across a variety of other physical stimuli. Microbubble-assisted ultrasound in head and neck cancer cells 10, nanodroplet-assisted FUS in fibrosarcoma xenografts 21, hypoxic conditions in breast cancer cells 14, irradiation and photodynamic therapy in prostate cancer cells 7,11 and UV radiation in colon cancer cells 26 have all been demonstrated to elicit EV release. The impact of elevated tumor-derived EVs is not immediately clear as the cargo contained within these EVs would largely dictate the direction in which they would tilt the tumor suppression/progression scale.

In order to further interrogate the potential role of GEVs released following FUS hyperthermia, we evaluated the proteomic repertoire of GEV isolates by mass spectrometry. Proteomic profiling revealed that GEVs exposed to FUS hyperthermia were proteomically distinct from their control counterparts. We observed that a number of markers associated with cancer progression and resistance were downregulated by GEVs following FUS hyperthermia. For instance, we noted significant downregulation of major vault protein (MVP) in FUS-exposed GEVs (p<0.0001); MVP is thought to be a miRNA-binding protein with a role in sorting miRNA to exosomes, is overexpressed in multidrug-resistant cancer cells 27,28 and has also been demonstrated to support glioblastoma (GBM) survival and migration 29. Studies have correlated calumenin (CALU) with tumor cell proliferation ability, with *CALU* transcript levels observed to be highly upregulated in GBMs 30,31. Consistent with conferral of a tumor-suppressive phenotype, we observed that CALU was significantly downregulated in GEVs treated with FUS (p<0.0001). We also observed that these GEVs downregulated annexin A6 (p=0.024), which has been shown to support tumor invasiveness and aggressiveness across multiple cancers 32,33. Finally, heat shock 70 kDa protein 5 (HSPA5), which has been correlated to tumor cell migration, invasiveness and clinical progression of triple negative breast cancer, was also significantly downregulated in GEVs following FUS (p<0.0001) 34.

We also noted some other interesting proteomic shifts following FUS hyperthermia. Consistent with elevation of EVs following FUS hyperthermia, we observed a significant increase in common EV markers - such as fibronectin (Fn1) (p<0.0001) - and cytoskeleton proteins on FUS^+^ GEVs. HSP90B1 (also known as GP96 or endoplasmin), which is mainly expressed in larger EVs, was significantly decreased in FUS-exposed GEVs 35 (p<0.0001). Interestingly, we noted a significant decrease in expression of calreticulin, an endoplasmic reticulum (ER) chaperone, by GEVs following FUS hyperthermia (p=0.0008). Studies have shown that such exclusionary markers as calreticulin, can be depleted in EVs isolated by differential ultracentrifugation and thereby serve as further confirmation of EV preparation purity 36,37. Finally, the observation of significant complement C5 upregulation (p=0.021) following FUS may serve as one potential linkage between hyperthermia and inflammation, as complement (e.g. C3, C5) has been linked to NF-κB signaling 38.

Interestingly, functional analysis of proteins significantly downregulated following FUS (Table 1) revealed that 18 of these hits are located in the ontological category of “extracellular exosome” (GO:0070062). As such, we cannot rule out the possibility that FUS exposure causes rearrangements in EV structure or a loss of cargo as a result of any such rearrangements. Taken together, the changes to GEV proteomic profile could reflect either direct impacts of FUS hyperthermia on cellular state or consequences of FUS exposure on cellular machinery regulating EV biogenesis, release and loading. As such, future studies should consider the direct impact of this FUS hyperthermia regimen on the proteomic profile of GL261-luc2 cells.

While our studies have important strengths, there are some limitations with respect to GEV isolation and proteomic analysis. First, the application of FUS necessitated the treatment of monolayered cells in an acoustically transparent, virtually hermetically closed system. Our utilization of cell culture chambers meeting these criteria inherently limited starting media volumes to <20 mL per chamber for EV isolation by differential ultracentrifugation. This, in turn, limited the amount of purified EVs available for molecular profiling. Future studies would benefit from the design and implementation of a platform whereby cells seeded in acoustically transparent chambers can be treated in a high-throughput manner in order to yield larger starting media volumes for EV isolation by differential ultracentrifugation and downstream biochemical assays. Moreover, *in vitro* EV characterization and functional studies are typically performed in the absence of serum-derived EVs, either through serum depletion or supplementation of growth medium with EV-depleted serum. Such precautions reduce “noise” attributed to interference between EVs from different species. The GL261-luc2 cell line failed to survive in the complete absence of FBS, so FUS hyperthermia experiments were conducted in the presence of 2% FBS. In light of potential bovine EV interference, mass spectrometry results were run against both murine and bovine databases to allow discernment between bovine and murine peptide sequences. This allowed an increased degree of certainty in identifying the tumor-derived EV proteins of murine origin that are reported herein. Any further inability to detect certain EV markers by mass spectrometry may have been due to their low stoichiometric expression.

To further probe the potential for FUS-exposed GEVs to influence inflammation, we designed an *in vitro* paradigm for evaluating the role of GEVs on IL-12p70 production by immortalized murine DC2.4 cells. By plating DC2.4 cells either in the presence or absence of GEVs, we were first able to determine that at baseline, GEVs dampen IL-12p70 production by DC2.4s in a GEV dose-dependent manner (Figure 5B). Interestingly, when GEVs were derived from FUS hyperthermia-exposed cells, IL-12p70 production was restored to a level near that of basal DC2.4 IL-12p70 production. While this restorative effect of FUS hyperthermia exposure on IL-12p70 production is largely consistent with the notion that FUS is lifting immunosuppressive barriers characteristic of naive GEVs or otherwise providing a favorable stimulus, it is not the only potential explanation for our observations. Alternatively, if FUS exposure bears consequence to the structure or payload of GEVs by causing GEV rearrangement, then the FUS^+^ GEVs could simply be rendered ineffective in reducing IL-12p70production by DC2.4. Future studies ought to investigate whether the distinct impact of FUS^+^ GEVs on DC2.4 cells is owing to active protection or inactive GEVs, perhaps by evaluating IL-12p70 production in response to mixed naïve and FUS-exposed GEVs.

We elected to explore the production of IL-12p70 in this preliminary investigation, as this cytokine plays a critical role in DC maturation and differentiation, as well as polarization of T helper cells toward an interferon-γ-producing type 1 (Th1) phenotype. We recognize that the scope of this *in vitro* study does not enable us to draw conclusions about the exact linkage between FUS^+^ GEVs and IL-12p70 production, nor does it permit extrapolation of such interactions to potential downstream potentiation of adaptive immunity since differentiation of CD4+ T-cells into Th1, Th2, or Th17 subtypes is not influenced by one particular cytokine alone. Future studies placing the effect of IL-12p70 production in the context of broader DC-mediated cytokine profiles will aid in determining the immunological impact of GEVs treated with FUS hyperthermia. In order to effectively enable further exploration of interactions between FUS and GEVs *in vivo*, we herein utilized a syngeneic luciferase-expressing glioma cell line capable of being readily translated into an immunocompetent mouse model with non-invasive bioluminescence imaging capability.

EVs are a powerful biological entity that play a critical role in cancer pathogenesis, tumor progression and tumor-immune interactions. Recent decades have seen an overwhelming resurgence in discoveries pertaining to the basic biology of EVs as well as to the exploitation of EVs as a clinical tool for cancer diagnosis and therapy 39. Among these roles, EVs shed by solid tumors are being explored heavily in the context of liquid biopsy as a non-invasive method for interrogating tumor biology, monitoring disease progression and evaluating treatment response 40. Indeed, a recent study has demonstrated that the mechanically disruptive effects elicited by FUS and nanodroplets can augment the release of tumor-derived EVs into the bloodstream of chicken embryos bearing fibrosarcoma xenografts 21. In this study, a variety of biomarkers (e.g. tumor-associated proteins, RNAs and miRNAs) with tumor-specific mutations reflective of tumor phenotype and aggressiveness were detectable within the payloads of these EVs. With this precedent in mind, cultivating a deeper understanding of how EVs can be leveraged to enable non-invasive liquid biopsy is of particular importance in the context of brain cancers, wherein minimally invasive biopsy is not possible. EVs can cross the blood brain barrier 41 and thus their profile in the peripheral circulation may serve as an attractive proxy for brain tumor progression and response to interventions such as FUS 42. This study demonstrates that FUS hyperthermia can have a profound impact on GEVs, lending to a potential mechanism by which FUS interfaces with the tumor-immune landscape as well as to the promise of leveraging GEV-associated biomarkers to monitor response to FUS.

## Figures and Tables

**Table 1:**
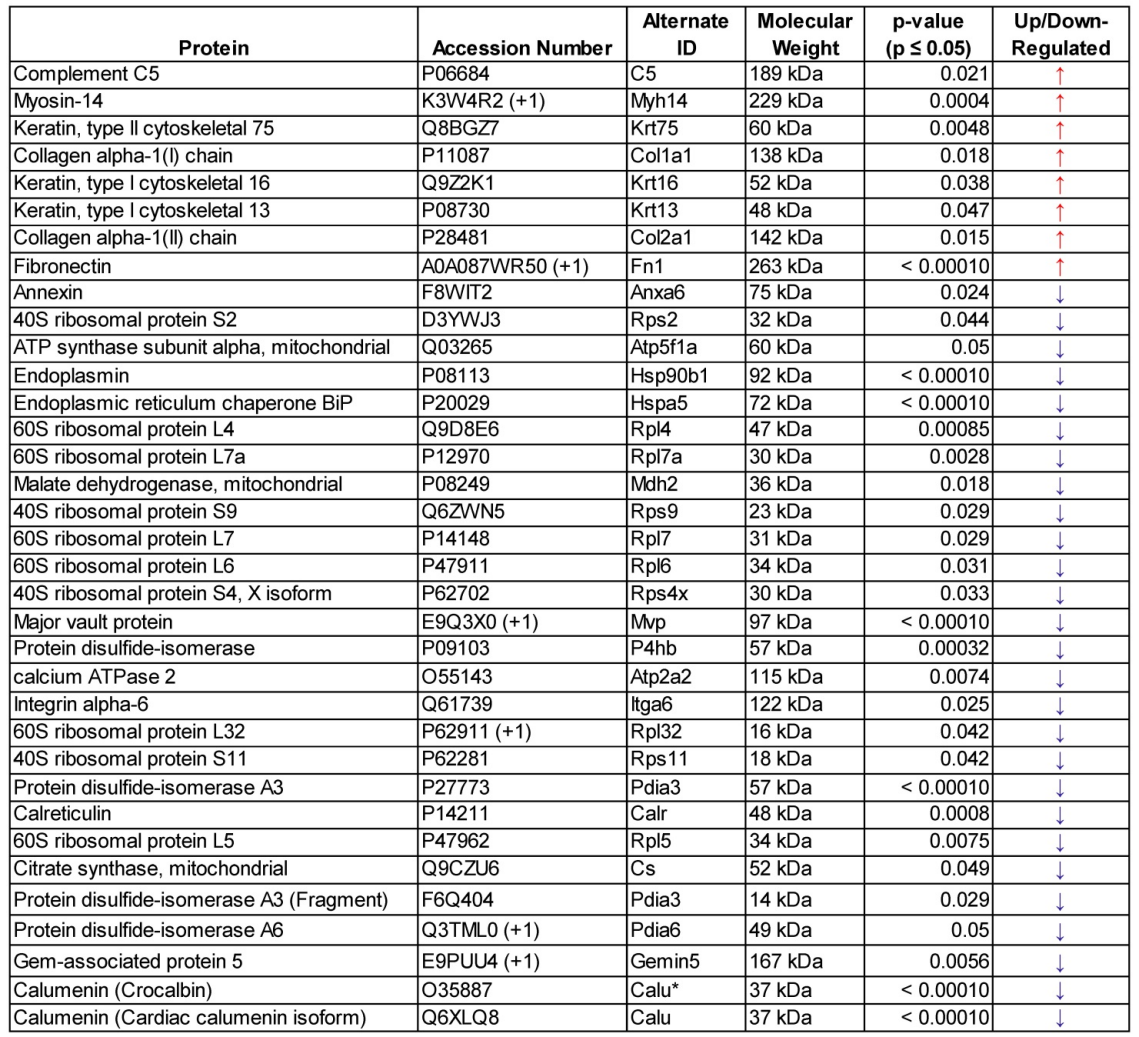
List of significantly regulated (p≤0.05) murine GEV proteins following FUS hyperthermia exposure - determined by mass spectrometry

## Abbreviations

EVs: extracellular vesicles
MVB: multivesicular body
FUS: focused ultrasound
GEVs): glioma-derived EVs
DCs): dendritic cells
SEM: standard error of the mean
NTA: nanoparticle tracking analysis
cryo-EM: cryogenic electron microscopy
Fn1: fibronectin
Myh14: myosin heavy chain 14
Krt6a: Keratin-6-alpha
Col1a1: collagen alpha-1(I)
Col2a1: collagen alpha-1(II)
C5: complement C5
Calu: calumenin
Hspa5: heat shock 70 kDa protein 5
Hsp90b1: endoplasmin or GP96
Calr: calreticulin
Itga6: integrin alpha-6
Anxa6: annexin
ELISA: enzyme-linked immunosorbent assay
IL-12p70: Interleukin-12, p70
GBM: glioblastoma
MVP: major vault protein
ER: endoplasmic reticulum
NF-κB: Nuclear factor kappa B
Th1: T helper type 1
